# Incidence and predictors of contralateral surgery after initial unilateral evacuation of bilateral chronic subdural hematomas: A population-based cohort study

**DOI:** 10.1016/j.bas.2026.106161

**Published:** 2026-07-01

**Authors:** Ali Buwaider, Edisson Nemer, John Anderberg, Alexander Fletcher-Sandersjöö, Jiri Bartek

**Affiliations:** a Department of Clinical Neuroscience, Karolinska Institute, Stockholm, Sweden; b Department of Neurosurgery, Karolinska University Hospital, Stockholm, Sweden; c Department of Neurosurgery, Rigshospitalet, Copenhagen, Denmark

**Keywords:** Chronic subdural hematoma, Bilateral, Recurrence, Predictors, Contralateral progression

## Abstract

**Introduction:**

The optimal surgical management of bilateral chronic subdural hematoma (bCSDH) remains debated, particularly whether and when to evacuate unilaterally or bilaterally at index surgery.

**Research question:**

This study aimed to determine the incidence and predictors of later contralateral surgery after initial unilateral evacuation of bCSDH.

**Material and methods:**

We conducted a population-based retrospective cohort study of adults (≥18 years) treated for bCSDH at Karolinska University Hospital (2006 – 2023). Standard surgical approach was single burr-hole craniostomy followed by 24-h subgaleal drainage. The primary outcome was evacuation of the initially non-operated hematoma within 6 months. Candidate predictors included preoperative clinical and radiological variables, as well as intraoperative treatment-related variables. Univariable analyses and backward stepwise multivariable logistic regression identified independent predictors.

**Results:**

Of 861 bCSDH cases, 401 underwent initial unilateral evacuation. Of these, later contralateral surgery was required in 46/401 (11%) at a median of 21 days (IQR 12 – 31) after the index procedure. Non-operated hematoma volume at index surgery was the only predictor of later contralateral evacuation (adjusted OR 1.01 per mL, p = 0.022), with a Nagelkerke pseudo-R^2^ value of 21%, and an AUC of 56%.

**Discussion and conclusion:**

In this population-based study, 11% of patients with bCSDH required contralateral surgery following unilateral evacuation, with a median time to surgery of 21 days. Non-operated hematoma volume at index surgery was statistically associated with later contralateral surgery, although its predictive performance was limited. These findings may help inform individualized postoperative surveillance strategies, particularly in patients with larger non-operated hematoma volumes.

## Highlights

• Largest reported cohort of bilateral chronic subdural hematomas to date.
• Risk of later contralateral CSDH surgery was 11%.
• Contralateral hematoma volume was associated with later hematoma progression.
• Contralateral hematoma volume may help guide postoperative surveillance.

## 1 Introduction

Chronic subdural hematoma (CSDH) is among the most common conditions in neurosurgery (Adhiyaman et al., 2002). With the increasing average life expectancy globally, and the rising use of antithrombotic agents, the incidence of CSDH is expected to grow (Balser et al., 2015). Despite its frequency, significant controversies remain regarding optimal management of CSDH (Zolfaghari et al., 2021; Duerinck et al., 2022; Solou et al., 2022). One notable controversy pertains to the management of bilateral CSDH (bCSDH), which accounts for approximately 25–30% of cases (Adhiyaman et al., 2002; Duerinck et al., 2022; Buwaider et al.). While surgical evacuation is the standard treatment, there is no consensus on whether both sides should be operated at the index procedure. Many surgeons evacuate only the more symptomatic or radiologically dominant side when the contralateral hematoma is small, reserving bilateral evacuation for cases with large collections on both sides. Between these two options is a grey zone of uncertainty, where the contralateral hematoma is neither negligible nor clearly in need of surgical drainage. In such cases, decisions are often guided by individual experience rather than evidence.

Current literature reports a 4 – 41% risk of subsequent surgery for the non-operated hematoma in unilaterally treated bCSDH (Takahashi et al., 2018; Zhang et al., 2020; Motiei-Langroudi et al., 2019; Shen et al., 2019; Scheichel et al., 2019; Andersen-et al., 2017; Fujitani et al., 2017; Yagi et al., 2025; Foppen et al., 2025). A second procedure is associated with higher complication rates, neurological deficits, prolonged hospitalization and increased costs (Takahashi et al., 2018; Zhang et al., 2020; Motiei-Langroudi et al., 2019; Shen et al., 2019; Scheichel et al., 2019; Andersen-et al., 2017; Fujitani et al., 2017). Identifying predictors of contralateral hematoma progression and determining optimal follow-up protocols are therefore important for improving postoperative care in this patient category.

To date, nine studies have investigated predictors of contralateral progression requiring surgery. Identified risk factors include contralateral hematoma volume, contralateral hematoma diameter, use of antithrombotic treatment, midline shift, and contralateral hematoma attenuation (Table 1). (Takahashi et al., 2018; Zhang et al., 2020; Motiei-Langroudi et al., 2019; Shen et al., 2019; Scheichel et al., 2019; Andersen-et al., 2017; Fujitani et al., 2017) However, these studies are limited by small sample sizes (analyzing between 22 and 136 hematomas), the lack of standardized treatment protocol, and inconsistent outcome reporting (Takahashi et al., 2018; Zhang et al., 2020; Motiei-Langroudi et al., 2019; Shen et al., 2019; Scheichel et al., 2019; Andersen-et al., 2017; Fujitani et al., 2017). Furthermore, no prior study has reported a cohort where single burr-hole craniostomy, followed by active subgaleal drainage, is standard protocol. Against this background, we aimed to determine the incidence and predictors of later contralateral evacuation after initial unilateral surgery for bCSDH using a large population-based cohort from a tertiary referral center with standardized treatment protocols and outcome assessment (Buwaider et al.).

**Table 1:** Summary of prior studies evaluating predictors of contralateral hematoma progression following unilateral evacuation of bilateral chronic subdural hematoma.

Author (year of publication, Country)	Unilaterally evacuated bCSDHs	Incidence of contralateral progression	Median time to contralateral surgery	Treatment	Outcome reporting	Reported predictors of contralateral surgery
Andersen-Ranberg et al. (Andersen- et al., 2017) (2017, Denmark)	136	12%	73 days	No standardized protocol^a^	No standardized reporting^b^	None
Fujitani et al. (Fujitani et al., 2017) (2017, Japan)	93	19%	46 days	Single BHC + passive subdural drain	Recurrence (6 mo); no standardized complications/function	Preoperative isodensity/hypodensity on T1 MRI of contralateral hematoma
Takahashi et al. (Takahashi et al., 2018) (2018, Japan)	22	21%	38 days	Single BHC + unspecified drainage	Recurrence (6 mo); no standardized complications/function	Contralateral hematoma volume
Scheichel et al. (Scheichel et al., 2019) (2018, Austria)	42	21%	28 days	Single/double BHC + unspecified drainage	No standardized reporting^b^	Contralateral hematoma diameter
						Ratio of operated to contralateral hematoma volume
Motiei-Langroudi et al. (Motiei-Langroudi et al., 2019) (2019, USA)	77	9.1%	58 days	No standardized protocol^a^	Recurrence (3 mo); no standardized complications/function	Contralateral hematoma volume
						Contralateral hematoma diameter
						Midline shift
Shen et al. (Shen et al., 2019) (2019, China)	53	32%	69 days	Single BHC + passive subdural drain	No standardized reporting^b^	Contralateral hematoma volume
Zhang et al. (Zhang et al., 2020) (2020, Singapore)	98	4.1%	31 days	No standardized protocol^a^	mRS for function + recurrence (6 mo); no standardized complications	Antithrombotic use
						Contralateral hematoma diameter
Yagi et al. (Yagi et al., 2025) (2025, Japan)	39	41%	Not reported	Single BHC + passive subdural drain	Recurrence (3 mo); no standardized complications/function	Contralateral hematoma volume
Foppen et al. (Foppen et al., 2025) (2025, Netherlands)	67	9.0%	32 days	Single BHC + unspecified drainage	No standardized reporting^b^	Small diameter of operated hematoma
						Contralateral hematoma diameter
Present study	401	11%	21 days	Single BHC + active subgaleal drainage	Standardized reporting of complications, function, and recurrence	Contralateral hematoma volume

Abbreviations: bCSDH, bilateral chronic subdural hematoma. BHC, burr-hole craniostomy. MRI, magnetic resonance imaging. mRS, modified Rankin Scale.

a lack of a standardized treatment protocol was defined as the absence of a clearly described surgical treatment strategy.

b lack of standardized outcome reporting was defined as the absence of established outcome measures for postoperative endpoints, including recurrence, complications, and functional status.

## 2 Material and methods

Adult patients (≥18 years) who were surgically treated for bCSDH at the Department of Neurosurgery at Karolinska University Hospital, Stockholm, Sweden, between January 1, 2006, and December 31, 2023, were included. Cases were identified using the NOMESCO code "AAD10" and retrieved from the hospital's surgical software Orbit (Evry Healthcare Systems, Solna, Sweden).

Exclusion criteria included CSDHs in arachnoid cysts, external hydrocephalus, or prior intracranial surgery within six months. Clinical data were retrieved from the electronic records software TakeCare (CompuGroup Medical Sweden AB, Farsta, Sweden), and imaging data were obtained from Sectra Picture Archiving and Communication System IDS7 (Sectra AB, Linköping, Sweden). The study followed STROBE guidelines for observational studies.

Given the retrospective design of the study and the mortality of many included patients during the study period, obtaining direct patient consent was not feasible and ethical approval was therefore provided by the regional ethical review board Stockholm EPN #2013/591–31/1 and #2017/247.

### 2.1 Treatment protocol

CSDH diagnosis was confirmed using computed tomography (CT) or, less commonly, magnetic resonance imaging (MRI), with perioperative confirmation. Anticonvulsants, corticosteroids, statins, and tranexamic acid were not part of routine management. The standard surgical technique throughout the 18-year study period was single burr-hole craniostomy, performed under local anesthesia, and followed by 24-h active subgaleal drainage (Sjåvik et al., 2017). In cases of multiple septations or inadequately liquefied hematomas, surgeons occasionally opted for a minicraniotomy.

The decision on laterality (unilateral vs bilateral evacuation) was determined based on symptoms and radiological findings. Hematomas deemed symptomatic or with clearly large volume or mass effect were evacuated. Smaller, asymptomatic, contralateral hematomas were left untreated. No specific size threshold was used to determine the need for bilateral evacuation. When both hematomas were deemed to warrant surgical intervention, bilateral evacuation was performed during the same operative session.

A single dose of antibiotics was routinely administered, and normal hemostasis was ensured preoperatively. Postoperative follow-up was performed either at the neurosurgical outpatient clinic or in primary care. For patients with bCSDH managed unilaterally, a follow-up CT scan was usually recommended to monitor the contralateral hematoma.

### 2.2 Variables

Preoperative variables included age, sex, presenting symptoms, Charlson Comorbidity Index (CCI) (Charlson et al., 1987), Glasgow Coma Scale (GCS) (Jain and Iverson, 2022), and the use of antithrombotic treatment. Detailed radiological analysis was performed on both the operated and non-operated hematomas. Radiological variables included hematoma attenuation (hypodense (<30 HU), isodense (30–60 HU), hyperdense (>60 HU), or mixed (a combination of hyperdense and isodense/hypodense)), diameter (maximum distance between the skull's inner layer and the brain surface), hematoma volume (calculated using the ABC/2 formula on the same slice) (Kothari et al., 1996), midline shift (greatest distance from the midline to the septum pellucidum) (Zanolini et al., 2022), ratio of diameters between the operated and non-operated hematomas, and hematoma architecture (according to Nakaguchi's classification) (Nakaguchi et al., 2001). Hematomas with multiple architectures were labeled as mixed, while those that did not match any classification were considered unstructured (Supplementary Fig. 1).

Postoperative variables included the Glasgow Outcome Scale (GOS) (Jennett and Bond, 1975) at discharge, as well as perioperative and postoperative complications occurring within 30 days, according to the Landriel Ibanez classification (Landriel et al., 2011). Other variables included ipsilateral hematoma recurrence requiring reoperation within six months of the initial surgery, length of hospitalization, and the follow-up plan chosen for each patient. The primary outcome of interest was contralateral hematoma requiring surgery following a unilateral evacuation of a bCSDH within six months. In cases where no contralateral procedure was required, the natural course of the non-operated hematoma was assessed when follow-up imaging was available.

### 2.3 Statistical analysis

Categorical data was presented as numbers (proportion). For continuous data, the Shapiro-Wilk test was used to assess data distribution. Normally distributed continuous data was presented as mean (± standard deviation), and non-normally distributed data as median (interquartile range). Between-group comparisons were performed using Mann-Whitney *U* test for continuous variables and the chi-square test for categorical variables. The cumulative risk of later contralateral hematoma evacuation over time was illustrated using a Kaplan-Meier curve. Predictors of contralateral hematoma progression requiring later evacuation were analyzed using univariable and multivariable backward stepwise logistic regression with variable selection based on p-value <0.05. Model performance was evaluated using Nagelkerke's pseudo-R^2^. A p-value <0.05 was considered statistically significant. All analyses were performed using R statistical software (R Foundation for Statistical Computing, Vienna, Austria).

## 3 Results

**Fig. 1:**
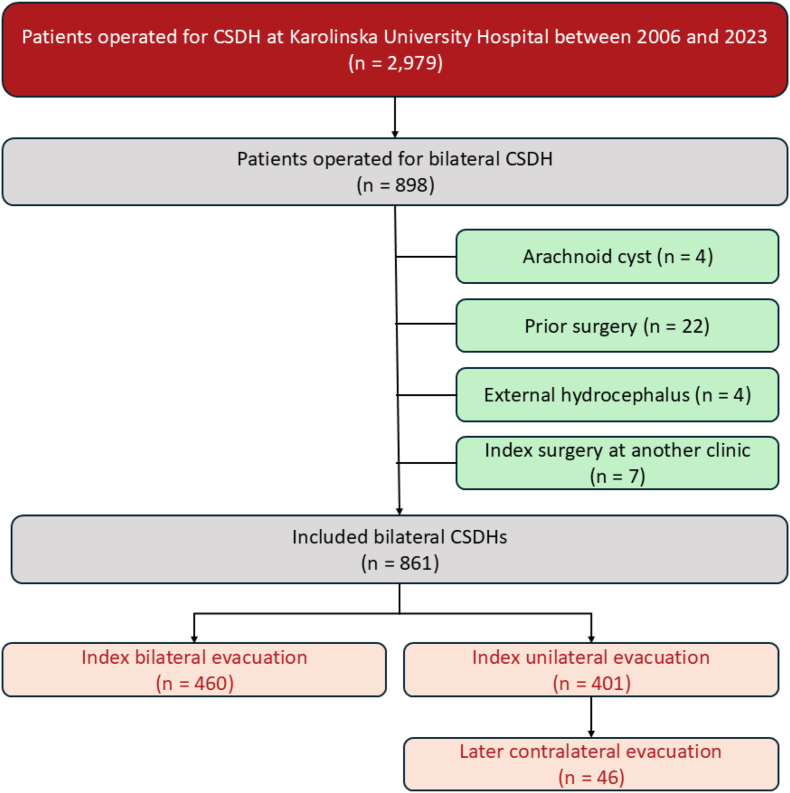
Flow-chart of the study population. *Abbreviation:* CSDH, chronic subdural hematoma.

Among the 2979 patients diagnosed with CSDH during the study period, 898 (30%) had bCSDH. Of these, 37 patients were excluded due to prior intracranial surgery within the past 6 months (n = 22), CSDH associated with arachnoid cysts (n = 4), or external hydrocephalus (n = 4). Seven patients were excluded since they had undergone the index surgery at another institution. As a result, 861 cases of bCSDH were included in the study. At the index procedure, 460 (53%) were treated bilaterally, and 401 (47%) were treated unilaterally (Fig. 1). Among the unilaterally treated bCSDH cases, 46 (11%) required later evacuation of the contralateral hematoma.

### 3.1 Baseline variables

The median age of patients was 76 years (IQR 69–83). Most patients were male (75%), with a median CCI of 1 (IQR 0–2). Most patients had some degree of disability but maintained a well-preserved level of consciousness at the time of admission, with a median GCS score of 15 (IQR 14–15). A significant portion (38%) were on antithrombotic treatment (Table 2).

**Table 2:** Baseline characteristics of patients with bilateral chronic subdural hematomas, stratified by unilateral versus bilateral evacuation at index surgery.

Variable	Overall bCSDH cohort (n = 861)	Index bilateral evacuation (n = 460)	Index unilateral evacuation (n = 401)	p-value
Age (years)	77 (69 – 83)	76 (69 – 83)	77 (69 – 83)	0.374
Female sex	217 (25%)	105 (23%)	112 (28%)	0.101
CCI score	1 (0 – 2)	1 (0 – 2)	1 (0 – 2)	0.108
Preoperative GCS	15 (14 – 15)	15 (14 – 15)	15 (14 – 15)	0.457
Preoperative symptoms				
Fall tendency	380 (44%)	225 (49%)	155 (39%)	**0.003**
Headache	321 (37%)	182 (40%)	139 (35%)	0.158
Paresis	333 (39%)	148 (32%)	185 (46%)	**<0.001**
Confusion	232 (27%)	130 (28%)	102 (25%)	0.393
Cognitive deficits	188 (22%)	103 (22%)	85 (21%)	0.734
Antiplatelet therapy	167 (19%)	91 (20%)	76 (19%)	0.825
Anticoagulant therapy	165 (19%)	83 (18%)	82 (20%)	0.419
Midline shift (mm)	4.0 (0 – 7.0)	2 (0 – 5)	7 (4 – 10)	**<0.001**
None	240 (28%)	203 (44%)	37 (9.2%)	**<0.001**
Thick hematoma				
Diameter (mm)	20 (16 – 24)	18 (15 – 22)	22 (17 – 26)	**<0.001**
Volume (mL)	111 (82 – 148)	101 (76 – 132)	127 (95 – 167)	**<0.001**
Attenuation				**<0.001**
Mixed	65 (7.5%)	10 (2.2%)	55 (14%)	
Hyperdense	4 (0.5%)	0 (0%)	4 (1.0%)	
Isodense	493 (57%)	299 (65%)	194 (48%)	
Hypodense	299 (35%)	151 (33%)	148 (37%)	
Nakaguchi				**<0.001**
Gradation	289 (34%)	176 (38%)	113 (28%)	
Homogenous	180 (21%)	104 (23%)	76 (19%)	
Laminar	22 (2.6%)	12 (2.6%)	10 (2.5%)	
Mixed	9 (1.0%)	7 (1.5%)	2 (0.5%)	
Separated	105 (12%)	54 (12%)	51 (13%)	
Trabecular	236 (27%)	102 (22%)	134 (33%)	
Unstructured	20 (2.3%)	5 (1.1%)	15 (3.7%)	
Thin hematoma				
Diameter (mm)	11 (8 – 15)	14 (11 – 16)	9 (6 – 12)	**<0.001**
Volume (mL)	53 (29 – 81)	76 (53 – 97)	30 (19 – 51)	**<0.001**
Attenuation				**<0.001**
Mixed	34 (3.9%)	14 (3.0%)	20 (5.0%)	
Hyperdense	11 (1.3%)	0 (0%)	11 (2.7%)	
Isodense	476 (55%)	272 (59%)	204 (51%)	
Hypodense	340 (40%)	174 (38%)	166 (41%)	
Nakaguchi				**<0.001**
Gradation	311 (36%)	193 (42%)	118 (29%)	
Homogenous	276 (32%)	115 (25%)	161 (40%)	
Laminar	18 (2.1%)	9 (2.0%)	9 (2.2%)	
Mixed	4 (0.5%)	4 (0.9%)	0 (0%)	
Separated	81 (9.4%)	51 (11%)	30 (7.5%)	
Trabecular	159 (19%)	83 (18%)	76 (19%)	
Unstructured	12 (1.4%)	5 (1.1%)	7 (1.7%)	
Ratio of hematoma volumes	0.63 (0.41 – 0.82)	0.78 (0.64 – 0.89)	0.41 (0.28 – 0.58)	**<0.001**
Surgical procedure				0.460
Burr-hole craniostomy	783 (91%)	418 (91%)	365 (91%)	
Minicraniotomy	78 (9%)	42 (9%)	36 (9.0%)	
Outcomes				
GOS at discharge	5 (4 – 5)	5 (4 – 5)	5 (4 – 5)	0.564
Days to discharge	2 (2 – 3)	2 (2 – 3)	2 (2 – 3)	0.084
Reoperation	109 (13%)	65 (14%)	44 (11%)	0.198
Days to reoperation	28 (17 – 45)	28 (19 – 46)	28 (19 – 46)	0.308
Complications within 30 days	84 (9.8%)	54 (11.7%)	30 (7.5%)	0.471
Ibanez 1	56 (6.5%)	36 (7.8%)	20 (5.0%)	
Ibanez 2	12 (1.4%)	6 (1.3%)	6 (1.5%)	
Ibanez 3	6 (0.7%)	4 (0.9%)	2 (0.5%)	
Ibanez 4	10 (1.2%)	8 (1.7%)	2 (0.5%)	
30-day mortality	29 (3.4%)	10 (2.5%)	19 (4.1%)	0.428

Values are presented as median (interquartile range) or number (percentage). Bold text in the p-value column indicates significance. Abbreviations: bCSDH, bilateral chronic subdural hematoma. CCI, Charlson Comorbidity Index. GCS, Glasgow Coma Scale. GOS, Glasgow Outcome Scale.

Median midline shift was 4 mm (IQR 0 – 7). 28% of cases had no midline shift, which could occur when both hematomas exerted a similar expansive effect. For the thick hematoma: median diameter was 20 mm (IQR 16 – 24) and median volume was 111 mL (IQR 82 – 148). The most common attenuation was isodense (57%), and most common Nakaguchi class was gradation (34%). For the thin hematoma: median diameter was 11 mm (IQR 8 – 15) and median volume was 53 mL (IQR 29 – 81). Most common attenuation was isodense (55%), and most common Nakaguchi class was gradation (36%). The median ratio between thick and thin hematomas was 0.63 (IQR 0.41 -0.82).

Surgery was primarily performed under local anesthesia (94%). Most patients underwent single burr-hole craniostomy (91%) followed by postoperative active subgaleal drainage (94%). Upon discharge, most patients achieved complete recovery, with a median GOS score of 5 (IQR 4–5), regardless of the laterality of surgery. The overall reoperation rate within six months was 13%. Complication rate was 9.8%, with Ibanez grade I complications being the most common (6.5%). The median hospitalization duration was 2 days (IQR 2–3).

### 3.2 Comparisons between index unilateral and bilateral evacuation

Patients undergoing index unilateral evacuation presented more frequently with paresis (46% vs 32%, p < 0.001), whereas a tendency to fall was more common among those treated with index bilateral evacuation (49% vs 39%, p = 0.003). The index bilateral group exhibited significantly less midline shift (median 2 mm vs 7 mm, p < 0.001), with 44% showing no shift at all. The median volume ratio between the smaller and larger hematoma was significantly higher in the index bilateral group (0.78 vs 0.41, p < 0.001), indicating more symmetric collections. In terms of density, hematomas in the index bilateral group were predominantly isodense, whereas those in the index unilateral group were more frequently hypodense (p < 0.001). Differences in hematoma architecture were also observed. In index bilateral patients, the architecture of operated and non-operated hematomas was quite similar, whereas in index unilaterally treated patients, the operated hematoma was significantly more trabecular compared to the non-operated contralateral hematoma (Table 2).

### 3.3 Incidence and predictors of later contralateral surgery

**Fig. 2:**
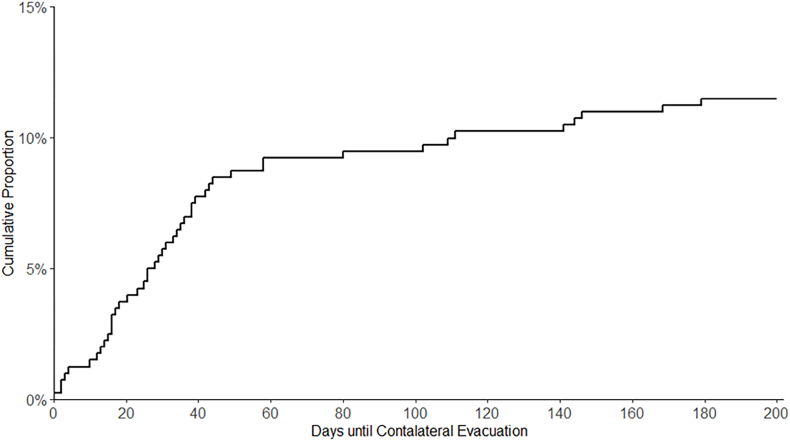
Cumulative risk of later contralateral hematoma evacuation following index unilateral evacuation of bilateral chronic subdural hematoma.

The rate of contralateral hematoma requiring later surgery was 11%, with the median time to surgery of 21 days (IQR 12–31) (Fig. 2).

Compared with patients who underwent unilateral evacuation of bilateral CSDHs, those who later required contralateral surgery had a longer cumulative length of hospital stay (median 4 days, IQR 4–6) and a higher recurrence rate (41% vs 8.2%); otherwise, clinical outcomes were comparable between the groups (Table 3).

**Table 3:** Characteristics of patients with bilateral chronic subdural hematomas undergoing initial unilateral evacuation, stratified by absence or presence of later contralateral surgery.

Variables	bCSDH patients with initial unilateral evacuation	
	Without later contralateral evacuation (n = 355)	With later contralateral evacuation (n = 46)
Age (years)	77 (69 – 83)	78 (72 – 83)
Female sex	101 (29%)	11 (24%)
CCI score	1 (0 – 2)	0.5 (0 – 2)
Preoperative GCS	15 (14 – 15)	15 (14 – 15)
Preoperative symptoms		
Fall tendency	142 (40%)	13 (28%)
Headache	113 (32%)	26 (57%)
Paresis	170 (48%)	15 (33%)
Confusion	89 (25%)	13 (28%)
Cognitive deficits	72 (20%)	13 (28%)
Antiplatelet therapy	72 (20%)	4 (8.7%)
Anticoagulant therapy	69 (19%)	13 (28%)
Midline shift (mm)	7 (4 – 10)	7 (3 – 9)
None	29 (8.2%)	8 (17%)
Operated hematoma		
Diameter (mm)	22 (17 – 26)	20 (15 – 25)
Volume (mL)	129 (95 – 170)	121 (79 – 146)
Attenuation		
Mixed	50 (14%)	5 (11%)
Hyperdense	4 (1.1%)	0 (0%)
Isodense	169 (48%)	25 (54%)
Hypodense	132 (37%)	16 (35%)
Nakaguchi		
Gradation	94 (27%)	19 (41%)
Homogenous	70 (20%)	6 (13%)
Laminar	8 (2.3%)	2 (4.3%)
Mixed	2 (0.6%)	0 (0%)
Separated	40 (11%)	11 (24%)
Trabecular	127 (36%)	7 (15%)
Unstructured	14 (3.9%)	1 (2.2%)
Non-operated hematoma		
Diameter (mm)	9 (6 – 11)	9 (6 – 12)
Volume (mL)	30 (19 – 50)	41 (15 – 62)
Attenuation		
Mixed	17 (4.8%)	3 (6.5%)
Hyperdense	9 (2.5%)	2 (4.3%)
Isodense	186 (52%)	18 (39%)
Hypodense	143 (40%)	23 (50%)
Nakaguchi		
Gradation	102 (29%)	16 (33%)
Homogenous	139 (39%)	22 (48%)
Laminar	8 (2.3%)	1 (2.2%)
Mixed	0 (0%)	0 (0%)
Separated	26 (7.3%)	4 (8.7%)
Trabecular	74 (21%)	2 (4.3%)
Unstructured	6 (1.7%)	1 (2.2%)
Ratio of hematoma volumes	0.40 (0.27 – 0.55)	0.50 (0.31 – 0.63)
Procedure		
Burr-hole craniostomy	327 (92%)	38 (83%)
Minicraniotomy	28 (8%)	6 (17%)
Outcomes		
GOS	5 (4 – 5)	5 (4 – 5)
Days to discharge^a^	2 (2 – 3)	4 (4 – 6)
Reoperation	29 (8.2%)	19 (41%)
Days to reoperation	24 (9.0 – 39)	36 (22 – 46)
Complications within 30 days^a^	29 (8.2%)	4 (8.7%)
Ibanez 1	20 (5.6%)	1 (2.2%)
Ibanez 2	5 (1.4%)	3 (6.5%)
Ibanez 3	2 (0.6%)	0 (0%)
Ibanez 4	2 (0.6%)	0 (0%)

Values are presented as median (interquartile range) or number (percentage). Abbreviations: bCSDH, bilateral chronic subdural hematoma. CCI, Charlson Comorbidity Index. GCS, Glasgow Coma Scale. GOS, Glasgow Outcome Scale.

a Outcomes of patients are shown cumulatively from both first and second surgery.

In the univariable logistic regression, significant predictors of later contralateral hematoma evacuation were larger initial non-operated hematoma volume (p = 0.022) and trabecular non-operated hematoma morphology (p = 0.019). In the subsequent backward stepwise regression analysis, which included all variables from the univariable analysis, non-operated hematoma volume remained the only independent predictor of later contralateral evacuation (OR 1.01, p = 0.022) (Table 4). The loss of significance of trabecular morphology in the final multivariable model suggests that its association with later contralateral surgery was largely explained by other radiological variables, particularly hematoma volume. The multivariable model had a Nagelkerke's pseudo-R^2^ value of 21%. An exploratory analysis using Youden's Index identified an optimal cut-off of 50 mL. However, its discriminatory performance was limited, with an AUC of 0.56 (95% CI, 0.45–0.66) (Supplementary File 2).

**Table 4:** Predictors of non-operated hematoma progression requiring evacuation.

Variable	Univariable logistic regression		Final step-down multivariable logistic regression Pseudo R^2^ = 21%	
	OR (95% CI)	p-value	OR (95% CI)	p-value
Age (years)	1.00 (0.97 – 1.03)	0.975	-	
Female sex	0.79 (0.37 – 1.57)	0.519	-	
CCI score	1.08 (0.93 – 1.45)	0.493	-	
Preoperative GCS	1.01 (0.83 – 1.24)	0.890	-	
Antiplatelets	0.37 (0.11 – 1.04)	0.069	-	
Anticoagulants	1.63 (0.79 – 3.20)	0.165	-	
Midline shift (mm)	0.95 (0.89 – 1.02)	0.189	-	
Contralateral hematoma diameter (mm)	1.02 (0.95 – 1.10)	0.528	-	
Contralateral hematoma volume (mL)	1.01 (1.00 – 1.02)	**0.022**	**1.01 (1.00**–**1.02)**	**0.022**
Contralateral hematoma attenuation				
Mixed	1.10 (0.24 – 3.60)	0.889	-	
Hyperdense	1.38 (0.20 – 5.80)	0.691	-	
Isodense	0.60 (0.31 – 1.15)	0.127	-	
Hypodense	Ref	Ref	-	
Contralateral hematoma Nakaguchi				
Gradation	0.92 (0.45 – 1.87)	0.838	-	
Homogenous	Ref	Ref	Ref	Ref
Laminar	0.79 (0.04 – 4.62)	0.827	-	
Separated	0.97 (0.27 – 2.80)	0.961	-	
Trabecular	0.17 (0.03 – 0.60)	**0.019**	-	
Unstructured	1.05 (0.05 – 6.58)	0.962	-	
Ratio between hematomas	3.38 (0.86 – 13.34)	0.082	-	
Procedure				
Burr-hole craniostomy	Ref	Ref	Ref	Ref
Minicraniotomy	2.15 (0.76 – 5.30)	0.116	-	

## 4 Discussion

In this large, consecutive, population-based cohort, we found that 11% of patients undergoing unilateral evacuation for bCSDH required later contralateral surgery. The median time to contralateral hematoma evacuation was 21 days, and the only independent predictor was the preoperative volume of the contralateral hematoma. These findings directly inform the ongoing debate on surgical laterality in bCSDH, where no clear guidelines exist for identifying patients at risk of contralateral progression.

### 4.1 Choice of laterality of surgery

In clinical practice, the decision between unilateral and bilateral evacuation is usually based on symptom lateralization and radiological findings. Patients with lateralized deficits, such as hemiparesis, are often treated with unilateral evacuation to relieve symptoms on the affected side (Ducruet et al., 2012). Similarly, when one hematoma is clearly larger, surgeons often choose unilateral evacuation, which was reflected in our data showing larger volumes, diameters, midline shift, and hematoma ratios in the unilateral group. Our strategy is in line with current literature, recommending evacuation of the symptomatic or larger hematoma (Greenberg, 2019). The lower attenuation and more frequent trabecular architecture of non-operated hematomas in unilaterally treated patients further suggest these hematomas are more chronic and may initially cause fewer symptoms, while bilaterally evacuated hematomas tend to present earlier and more acutely (Kwon et al., 2022).

### 4.2 Incidence of later contralateral evacuation

Sample sizes across the current nine studies range from 22 to 136 patients (Takahashi et al., 2018; Zhang et al., 2020; Motiei-Langroudi et al., 2019; Shen et al., 2019; Scheichel et al., 2019; Andersen-et al., 2017; Fujitani et al., 2017; Yagi et al., 2025; Foppen et al., 2025). This study presents therefore the largest reported cohort of bCSDHs to date. Reported risks of contralateral progression range from 4% to 41%, with median times to surgery ranging between 31 and 73 days (Takahashi et al., 2018; Zhang et al., 2020; Motiei-Langroudi et al., 2019; Shen et al., 2019; Scheichel et al., 2019; Andersen-et al., 2017; Fujitani et al., 2017; Yagi et al., 2025; Foppen et al., 2025) (Takahashi et al., 2018; Zhang et al., 2020; Motiei-Langroudi et al., 2019; Shen et al., 2019; Scheichel et al., 2019; Andersen-et al., 2017; Fujitani et al., 2017; Yagi et al., 2025; Foppen et al., 2025). However, these findings should be interpreted in the context of center-specific surgical protocols. Among the nine studies, three did not report a standardized treatment protocol (Zhang et al., 2020; Motiei-Langroudi et al., 2019; Andersen-et al., 2017); three did not specify standard drainage type (Takahashi et al., 2018; Scheichel et al., 2019; Foppen et al., 2025); and the remaining three described standard protocols of single burr-hole craniostomy followed by passive subdural drainage, rather than active subgaleal drainage (Shen et al., 2019; Fujitani et al., 2017; Yagi et al., 2025). The meta-analysis by Foppen et al. (2025), which pooled current nine studies, reported an overall incidence of 14%, which is comparable to the 11% observed in our cohort. Notably, our findings reflect a different standardized treatment strategy. This distinction is important, as it provides clinically relevant risk estimates for centers employing similar management protocols and may help guide postoperative surveillance strategies.

### 4.3 Contralateral hematoma volume

Contralateral hematoma volume has been consistently recognized as a significant predictor in studies where it was examined (Takahashi et al., 2018; Motiei-Langroudi et al., 2019; Shen et al., 2019). In studies where it was not explicitly examined, non-operated hematoma diameter was instead reported as a significant predictor (Zhang et al., 2020; Scheichel et al., 2019), reinforcing the importance of hematoma size. Our analysis confirmed that hematoma size is the primary driver of contralateral progression. We identified a possible inflection point at approximately 50 mL. However, this threshold must be interpreted with caution, due to a poor discriminatory power (AUC = 0.56). Even though a threshold of 50 mL cannot be recommended as a clinically reliable cutoff for prophylactic surgery, a larger contralateral volume in bCSDH should be taken into account when planning radiological surveillance. Management should be individualized on a case-by-case basis, with surgeons maintaining readiness for later contralateral evacuation if the non-operated hematoma has a large volume.

In the pooled meta-analysis by Foppen et al. (2025), volumes of both operated and non-operated hematomas were identified as significant independent risk factors. A common concern in the management of bCSDH is that evacuating a large ipsilateral hematoma creates a sudden 'pressure sink', hypothetically facilitating rapid expansion of the contralateral collection due to the swift reduction in intracranial pressure (Motiei-Langroudi et al., 2019). However, our findings challenge this hemodynamic theory. We found no significant association between the volume of the evacuated hematoma and the risk of contralateral progression. This suggests that contralateral expansion is likely driven by the intrinsic biological properties of the non-operated hematoma itself, rather than the mechanical volume reduction performed during the index surgery.

### 4.4 Strengths

To our knowledge, this is the only study reporting later contralateral surgery for bCSDHs treated with single burr-hole craniostomy followed by subgaleal drainage in a standardized manner. It represents the largest cohort of bCSDHs reported to date. The study provides population-based results as it was derived from a high-volume tertiary referral center, one of six neurosurgical centers in the country, with a catchment area of 2.5 million people (25% of the national population). Furthermore, this is the only study to report complications, recurrence rates and postoperative function using standardized outcome measures, enhancing the comparability and generalizability of the findings to other centers employing similar treatment protocols.

### 4.5 Limitations

This single-population study may not fully represent other populations, especially given Sweden's high life expectancy. (Life expectancy remains highest in Halland and County) While designed with standardized outcome measures, the study is subject to retrospective limitations such as selection bias and misclassification. Even though volume was significantly associated with higher risk of later contralateral evacuation, a discriminatory threshold to justify index bilateral evacuation could not be identified with this current cohort. Moreover, hematoma volume was estimated using the ABC/2 formula. Previous studies have demonstrated that this method can serve as a reasonable substitute for computer-based segmentation-based volumetric tools (Won et al., 2018; Fletcher-et al., 2024; Drake et al., 2025). However, retrospective analyses suggest that its accuracy may be limited. An analysis by Sucu et al. (2005) showed that applying the ABC/2 formula using maximum length and width measurements may provide more accurate volume estimation compared to same CT slice measurements. Although some calculated volumes may appear large, particularly for hematomas with volumes exceeding 100 mL, such values are plausible given the crescentic configuration and broad hemispheric spread of CSDH (Nouri et al., 2021; Kolias et al., 2014).

It should be noted that death during follow-up represented a competing event for the primary outcome. Since deaths were treated as censored observations in the Kaplan–Meier analysis, the cumulative incidence of contralateral surgery may have been slightly overestimated. However, given the large population and the relatively low 30-day mortality (4.1%), the impact on the overall results is likely limited.

Given the study's aim, we chose logistic regression with backward stepwise selection to identify predictors of later contralateral surgery and to present odds ratios that are easily interpretable for clinical practice. We acknowledge that alternative approaches, such as penalized regression methods, may offer advantages in terms of shrinkage, reduced overfitting, and improved model stability, particularly in the presence of multiple correlated predictors. However, these methods were not implemented in the current analysis, as the study was not designed as a predictive modeling framework.

## 5 Conclusion

In this population-based cohort where single burr-hole craniostomy followed by active subgaleal drainage is standard treatment, 11% of operated bCSDHs required later contralateral surgery following unilateral evacuation, typically within three weeks (median 21 days). Statistically, contralateral hematoma volume was the only independent predictor of later contralateral evacuation. Postoperative surveillance of a non-operated contralateral hematoma should be individualized on a case-by-case basis, and surgeons should anticipate the possible need for delayed contralateral evacuation in patients with large contralateral hematoma volume.

## Disclosures

The authors have no personal, financial, or institutional interest in any drugs, materials, or devices described in this article.

## Data availability

Data is provided from the corresponding author upon reasonable request.

## Authors' contributions

Conceptualization: AFS, JB. Methodology: AFS, JB. Data collection: AB, EN, JA, AFS. Analysis and interpretation: AB, AFS, JB. Writing of manuscript draft: AB, AFS, JB. Review and editing of manuscript: All authors. Final approval of manuscript: All authors. Study supervision: AFS, JB.

## Ethics approval

The study was approved by the regional ethical review board who waived the need for informed consent (Stockholm EPN #2013/591–31/1 and #2017/247).

## Funding

This research did not receive any specific grant from funding agencies in the public, commercial, or not-for-profit sectors.
