# Supplementary material for: Incidence and predictors of contralateral surgery after initial unilateral evacuation of bilateral chronic subdural hematomas: A population-based cohort study
Source: Brain Spine. 2026 Jul 1;6:106161. doi: 10.1016/j.bas.2026.106161 (PMC13355419; doi:10.1016/j.bas.2026.106161)
Supplement: Multimedia component 1 [file mmc1.docx]

**Supplementary Fig. 1. Radiological measurements.**


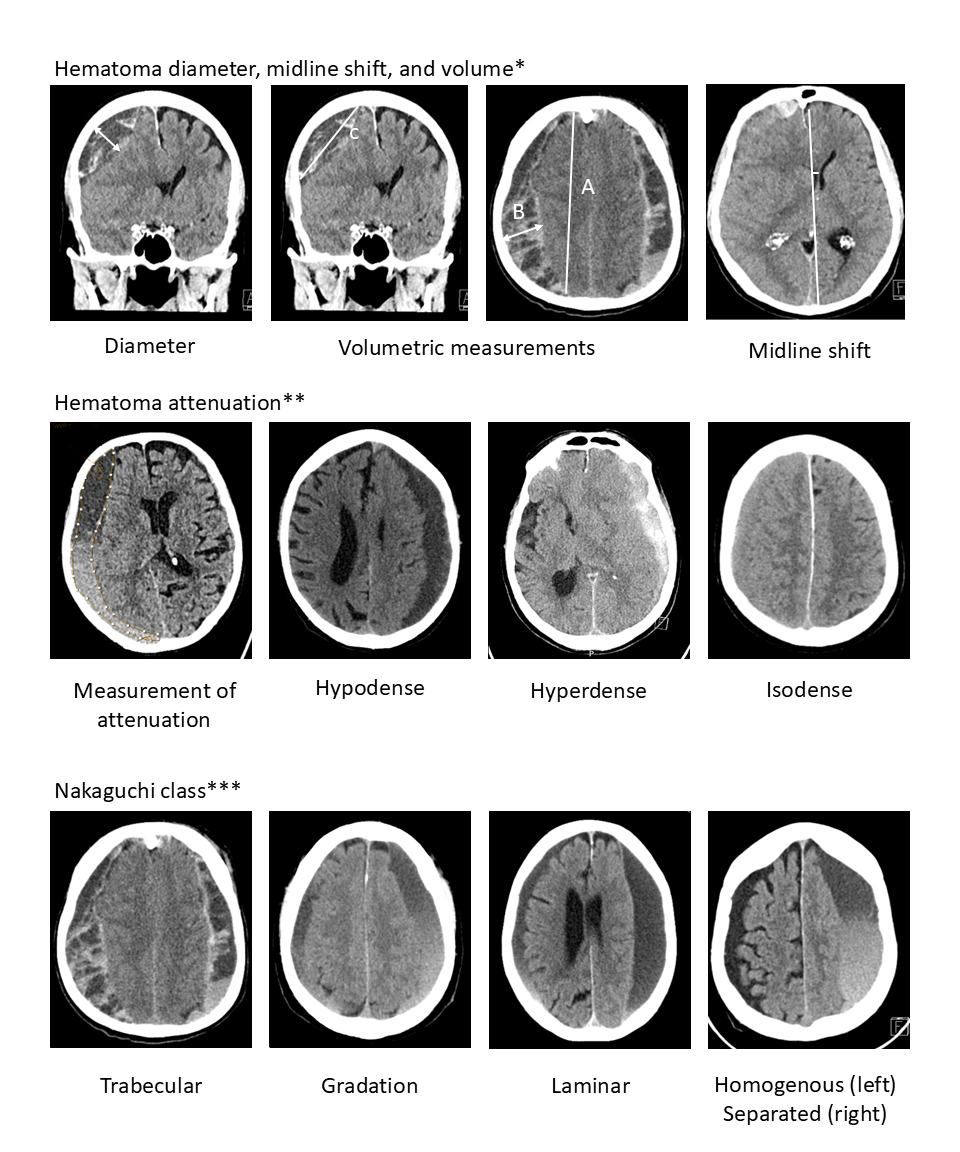


* Hematoma diameter was defined as the maximum distance from the inner table of the skull to the brain surface. Midline shift was measured as the greatest deviation from the midline, defined by a line connecting the anterior and posterior attachments of the falx cerebri. Hematoma volume was calculated using the ellipsoid formula (A × B × C / 2).

** Hematoma attenuation was assessed by calculating the mean Hounsfield unit after manually delineating the entire hematoma on an axial CT slice.

*** Hematoma architecture was classified according to the Nakaguchi classification.
