# Supplementary material for: Incidence and predictors of contralateral surgery after initial unilateral evacuation of bilateral chronic subdural hematomas: A population-based cohort study
Source: Brain Spine. 2026 Jul 1;6:106161. doi: 10.1016/j.bas.2026.106161 (PMC13355419; doi:10.1016/j.bas.2026.106161)

**Supplementary Fig. 2.** Optimal cut-off determination and diagnostic performance of the independent variable. The left panel shows the distribution of the independent variable stratified by outcome class, with the optimal cut-off indicated. The right panel displays the receiver operating characteristic curve illustrating the relationship between sensitivity and 1 − specificity across thresholds.


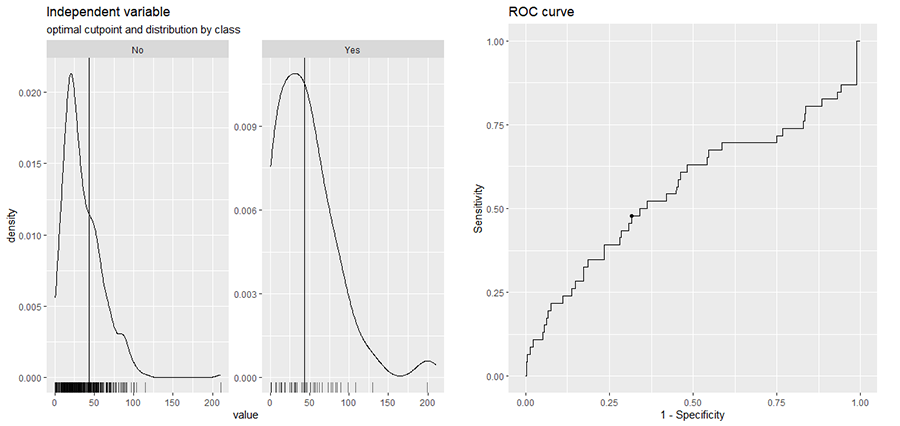

Supplement: Multimedia component 2 [file mmc2.docx]
